# Comparative efficacy of aerobic exercise and mind-body practices in improving sleep quality and psychological distress among elderly breast cancer patients: a systematic review

**DOI:** 10.3389/fonc.2026.1798402

**Published:** 2026-03-25

**Authors:** Cai Cui, Wei Liang, Xin Liu

**Affiliations:** 1 Department of Arts and Physical Education, Henan Technical College of Construction, Zhengzhou, China; 2 College of Physical Education, Yangzhou University, Yangzhou, China

**Keywords:** aerobic exercise, breast cancer, mind-body practice, psychological distress, sleep quality

## Abstract

Elderly breast cancer patients often face severe sleep disturbances and psychological distress due to the disease itself and its treatment process, significantly reducing their quality of life. In recent years, non-pharmacological interventions, represented by aerobic exercise and mind-body practices, have demonstrated great potential in improving the physical and mental health of patients. This paper aims to systematically review existing literature and compare the effects of aerobic exercise and mind-body practices on sleep quality and psychological distress in elderly breast cancer patients. The review focuses on analyzing the similarities and differences between the two intervention approaches in improving sleep architecture, alleviating psychological stress including anxiety and depression, and their associated physiological mechanisms. By synthesizing the latest evidence from clinical trials and intervention studies, this paper explores the clinical application value of these two intervention strategies, identifies current research limitations, and suggests future research directions. This aims to provide theoretical foundations and practical guidance for developing individualized, precision rehabilitation plans tailored to elderly breast cancer patients.

## 1 Introduction

With advances in breast cancer diagnosis and treatment technologies, patient survival rates have significantly improved. This has increasingly shifted clinical focus to post-treatment quality of life, particularly among elderly patients (1).

Globally, breast cancer incidence continues to rise, with projections indicating 3.2 million new cases and 1.1 million deaths worldwide by 2050, making it the most common cancer globally. Notably, while this review focuses on elderly patients, breast cancer incidence among younger women is also increasing, underscoring the necessity of providing age-appropriate survivorship care throughout the entire lifespan (2).

Within this population, sleep disturbances and psychological distress, anxiety and depression are highly prevalent. These issues not only severely impact the recovery progress and life satisfaction of patients, but may also exacerbate other cancer-related symptoms through complex psychophysiological mechanisms, creating a vicious cycle (3, 4). Research indicates that poor sleep quality correlates with higher levels of depression, fatigue, and poorer quality of life, with these effects potentially persisting for months or even years after treatment concludes (3). Psychological distress, particularly anxiety and depression, exhibits high prevalence among breast cancer patients. A screening study of young breast cancer survivors found that over one-fifth of participants met criteria for major depressive disorder, with these symptoms closely linked to anxiety, fatigue, insomnia, and other issues (4).Notably, a bidirectional relationship exists between psychological distress and physical symptoms. Patients with a history of psychological distress reported higher levels of pain, fatigue, sleep difficulties, and poorer self-rated health both before and after treatment (5). Furthermore, fatigue, sleep disturbances, anxiety, and depression often cluster together, mutually reinforcing each other and collectively impairing cognitive function and quality of life (6, 7). The existence of these symptom clusters often limits the effectiveness of interventions targeting a single symptom. Therefore, it is necessary to explore comprehensive intervention strategies that can simultaneously improve multiple symptoms.

Given the potential side effects and dependency associated with drug therapies, non-pharmacological interventions have garnered significant attention for their safety and multifaceted health benefits (8). Among these, aerobic exercise and mind-body practices represent two crucial non-pharmacological approaches. Walking, jogging, and cycling are common forms of aerobic exercise that have been proven to effectively improve cardiopulmonary function, reduce fatigue, and enhance overall health in cancer survivors (9). For instance, a study involving overweight or obese breast, prostate, and colorectal cancer survivors found that a 16-week circuit interval aerobic and resistance training intervention significantly improved self-reported sleep quality, and this improvement was significantly associated with reduced insulin resistance (9). Even during exceptional periods like the COVID-19 pandemic, a 16-week online home-based exercise intervention demonstrated significant improvements in sleep quality, reduced sleep latency, and increased sleep duration and efficiency among breast cancer patients (10). On the other hand, mind-body practices, such as meditation, yoga, Tai Chi, and hypnotherapy focus on alleviating stress, improving mood, and promoting relaxation by regulating mind-body connections (11). A randomized controlled trial evaluated the effects of a group intervention combining self-care and self-hypnosis on post-treatment cancer patients, and found it significantly improved fatigue, sleep, and emotional distress (11). Furthermore, cognitive behavioral therapy for insomnia has been shown to effectively reduce fatigue in cancer survivors, with this improvement largely mediated through alleviating insomnia symptoms (12).

Beyond standard multidisciplinary integrated care involving surgery, chemotherapy, radiotherapy, and endocrine therapy, the roles of various health professionals have been increasingly recognized as crucial in comprehensive cancer care. Psychologists and physical therapists play particularly vital roles in the later stages of treatment, collaborating with patients to address the multifaceted consequences of cancer and its therapies (13).Maintaining optimal physical and mental activity throughout cancer treatment is crucial for achieving positive therapeutic outcomes and enhancing women’s well-being (14).

Although aerobic exercise and mind-body practices have each demonstrated potential in improving specific symptoms among cancer patients, systematic summaries and evaluations of their direct comparative efficacy in enhancing sleep quality and alleviating psychological distress among elderly breast cancer patients remain lacking (15). Existing research suggests that breast cancer survivors across different age groups may face distinct psychosocial challenges. For instance, younger survivors may report more severe fatigue, loneliness, daytime sleepiness, and stress (15). Therefore, for the specific elderly population, clarifying the functional characteristics and relative advantages of different exercise modalities is crucial for developing individualized, precision non-pharmacological intervention plans. This study aims to systematically evaluate and compare the effects of aerobic exercise versus mind-body practices on sleep quality and psychological distress in elderly breast cancer patients, based on the latest clinical research evidence. This systematic review seeks to provide more targeted scientific evidence for clinical practice, thereby optimizing patient management and enhancing their long-term quality of life.

## 2 Current status of sleep disorders and psychological distress in elderly breast cancer patients

### 2.1 Impact of breast cancer treatments on sleep

Various breast cancer treatments including surgery, chemotherapy, radiotherapy, and endocrine therapy pose significant challenges to sleep quality, resulting in persistently high prevalence rates of sleep disorders. A systematic review indicates that the overall reported rate of sleep disorders among patients undergoing breast cancer treatment is approximately 60% (16). Specifically, using the Pittsburgh Sleep Quality Index (PSQI) for assessment, the average prevalence of poor sleep quality (PSQI > 5) following surgery, chemotherapy, radiotherapy, and endocrine therapy was 63%, 62%, 64%, and 57%, respectively (16). These data indicate that sleep problems are a common concern for breast cancer patients regardless of treatment modality. Notably, chemotherapy is the most extensively studied treatment modality. Among studies evaluating chemotherapy, 62% reported a significant increase in treatment-related sleep disturbances (16). An observational study involving 245 breast cancer patients further confirmed that women undergoing chemotherapy exhibited more severe insomnia symptoms and poorer sleep quality, with an increased number of chemotherapy cycles correlating with worsening insomnia symptoms (17). These treatments not only directly disrupt sleep but also create a vicious cycle with sleep disturbances through their side effects. For instance, treatment-related pain and fatigue are key factors exacerbating sleep disorders. A cross-sectional study found that breast cancer patients with sleep disorders were more likely to experience severe pain (18). Pre-radiotherapy sleep disturbances were also significantly associated with increased pain during treatment (19). Furthermore, cancer-related fatigue, which is closely intertwined with sleep disorders, is highly prevalent among breast cancer patients (20). This mutually reinforcing relationship between treatment side effects and sleep disorders severely impacts quality of life. Research indicates that sleep disturbances are associated with a significant deterioration in quality of life, affecting both daily functioning and symptom intensity (17). Therefore, systematically identifying and addressing sleep issues throughout the breast cancer care continuum is crucial to breaking this vicious cycle and improving overall health outcomes.

### 2.2 Manifestations of psychological distress in elderly breast cancer patients

Significant psychological distress is prevalent among elderly breast cancer patients, presenting in diverse and complex forms. Multiple studies indicate that anxiety, depression, and mood swings are the most common psychological symptoms among this patient group (21). A cross-sectional study of elderly cancer patients in China found that approximately 29.7% of participants exhibited high levels of psychological distress, often accompanied by poorer quality of life (21). Notably, while breast cancer patients tend to experience relatively milder depressive symptoms compared to other cancer types, anxiety symptoms remain particularly prominent (22). This psychological distress not only affects patients’ emotional states but also significantly reduces treatment adherence and quality of life, creating a vicious cycle.

A complex bidirectional relationship exists between psychological distress and sleep disorders. On one hand, persistent psychological stress disrupts sleep rhythms, leading to difficulties in falling asleep and maintaining sleep (21). On the other hand, reduced sleep quality exacerbates anxiety and depressive symptoms, further intensifying the vicious cycle of symptom progression. This interaction is particularly pronounced among elderly breast cancer patients, as older adults inherently possess more fragile sleep architecture (22). Studies further indicate that patients with specific demographic characteristics, such as being female, living alone, having higher education levels, or bearing financial burdens, appear more susceptible to psychological distress (21).

Additionally, elderly breast cancer patients face unique psychological challenges. Compared to younger patients, they must cope not only with the shock of a cancer diagnosis but also with multiple stressors associated with aging (23).Some studies indicate that the concerns of elderly patients about disease prognosis, fears of treatment side effects, and anxieties about family responsibilities may all contribute to increased psychological burden (24). Notably, the manifestations of psychological distress may vary across cultural contexts, suggesting the need for individualized psychological intervention strategies (21). Overall, psychological distress, as a common comorbid symptom among elderly breast cancer patients, warrants sufficient attention from clinicians and requires targeted intervention measures.

### 2.3 The reciprocal relationship between sleep disorders and psychological distress

A complex bidirectional relationship exists between sleep disorders and psychological distress, with multiple studies demonstrating a significant positive correlation between the two. A cross-sectional study of Iranian adolescents found that sleep hygiene partially mediated the association between gaming disorder (GD) and depressive symptoms, suggesting poor sleep habits may exacerbate depression in GD adolescents (25). Similarly, Mendelian randomization studies provide genetic evidence linking depression to increased sepsis risk, while post-traumatic stress disorder (PTSD) correlates with intensive care requirements for sepsis, suggesting psychological distress may impact physical health through neuroendocrine or inflammatory pathways (26). In families with children on the autism spectrum (ASD), parental depressive symptoms significantly correlated with childhood sleep disruption, indicating sleep problems may amplify psychological burdens through family interactions (27).

The underlying mechanisms may involve multisystem interactions. Neuroendocrine dysregulation is a central component, where prolonged psychological distress can lead to hyperactivation of the hypothalamic-pituitary-adrenal (HPA) axis, disrupting cortisol secretion rhythms and subsequently impairing the sleep-wake cycle. A study on COVID-19 patients demonstrated that short-term mindfulness meditation significantly improved sleep quality and reduced depression scores by enhancing mindfulness levels, suggesting psychological interventions may regulate autonomic nervous function to improve sleep (28). Inflammatory responses represent another critical pathway. Clinical data from trigeminal neuralgia patients indicate that sleep disturbances and obsessive thoughts are significantly correlated with Visual Analogue Scale (VAS) pain scores and Self-Rating Depression Scale (SDS) scores. suggesting chronic pain may influence sleep and mood through pro-inflammatory cytokine release (29). Surveys of atopic dermatitis patients further confirm that 73% experience poor sleep quality, with more severe sleep disturbances among those at risk for depression. This indicates skin itching and psychological distress may form a vicious cycle via Th2-type immune responses (30).

This interplay exhibits specificity across populations. A study of Chinese psychiatric nurses found significant correlations between psychological distress and negative coping styles (r=0.266) and sleep quality (r=0.532), with sleep quality partially mediating (37.97%) the relationship between negative coping and psychological distress (31). Studies on pediatric allergic diseases indicate that allergic rhinitis correlates with multiple sleep disturbances including irregular sleep patterns, morning fatigue, with sleep issues mediating the association between allergic conditions and psychological distress (32). For patients with somatic pain, chain mediation analysis confirmed a sequential mediating role of psychological distress and sleep disorders between physical pain and temporomandibular joint pain disorders, highlighting the “pain-emotion-sleep” triadic relationship (33). Collectively, these findings indicate that the interaction between sleep disorders and psychological distress is moderated by multiple factors including individual disease status, coping strategies, and social support, necessitating comprehensive intervention strategies.

## 3 Effects of aerobic exercise on sleep and mental health in elderly breast cancer patients

### 3.1 Physiological mechanisms of aerobic exercise

**Figure 1:**
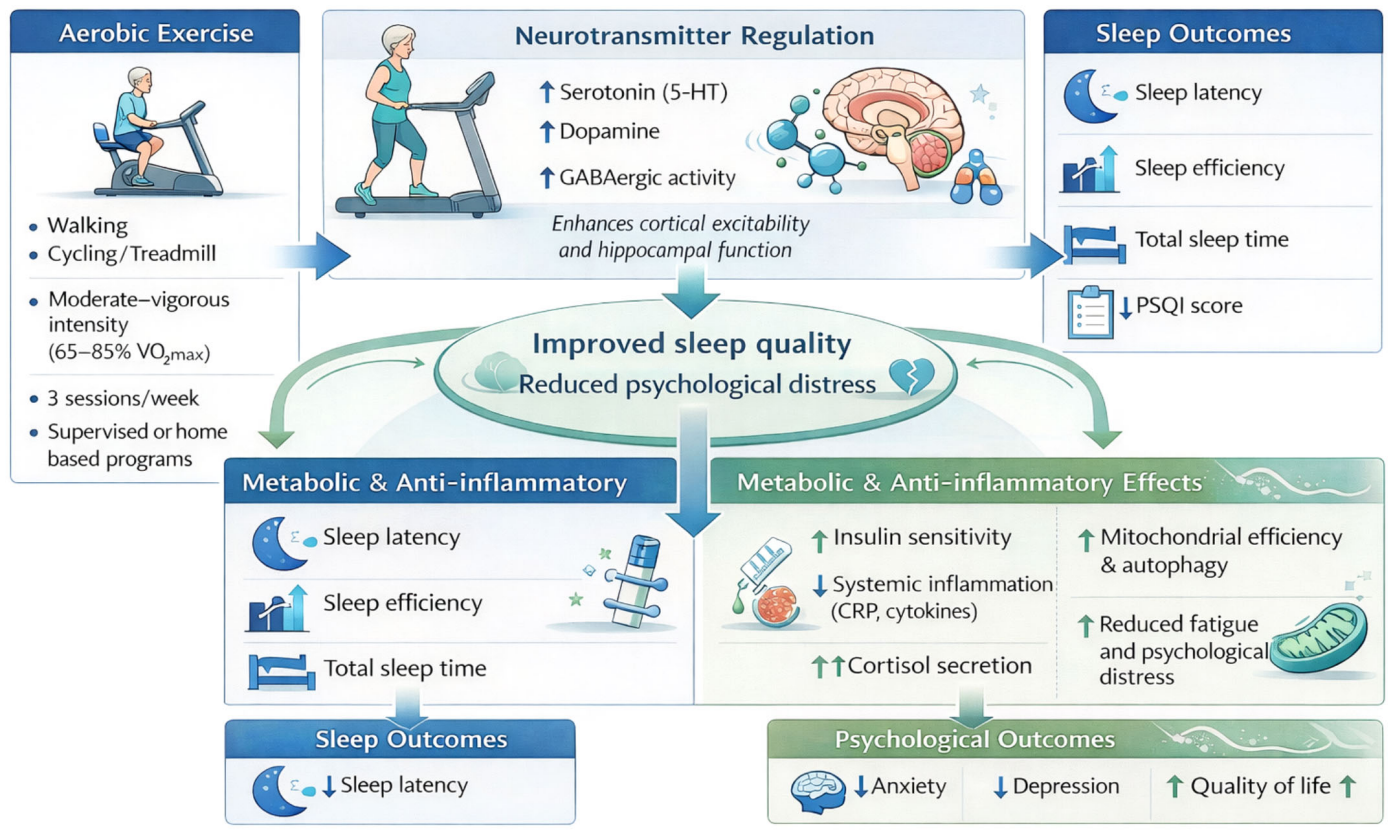
Mechanisms by which aerobic exercise improves sleep quality and psychological health in older breast cancer patients.

The physiological mechanisms underlying aerobic exercise’s improvement of sleep quality and psychological distress are multifaceted, primarily involving core pathways such as neurotransmitter regulation, metabolic balance, and inflammation control. First, aerobic exercise significantly modulates neurotransmitter levels in the central nervous system, particularly serotonin and dopamine, thereby directly influencing sleep architecture and emotional states. Research indicates that even a single aerobic session can regulate cortical excitability, a process linked to changes in gamma-aminobutyric acid (GABA) system activity. GABA is the primary inhibitory neurotransmitter in the CNS, crucial for promoting sleep and alleviating anxiety (34). A cross-over study in healthy subjects further confirmed that a single aerobic session not only enhances motor sequence learning ability but also increases cortical excitability. Moreover, changes in cortical excitability, particularly reduced short-term intracortical inhibition, correlating with the degree of improvement in motor learning, this suggests exercise influences neuroplasticity by modulating GABAergic activity, with such neuroplastic changes forming the basis for cognitive and emotional improvements (34). Furthermore, regular aerobic exercise has been shown to strengthen functional connectivity between the hippocampus and cortex, an enhancement beneficial for emotional regulation and stress response (35). These neurophysiological adaptations collectively constitute the neurobiological basis for efficacy of aerobic exercise in improving sleep and mood disorders, as illustrated in **Figure 1**.

Second, aerobic exercise indirectly yet effectively alleviates fatigue and psychological stress by promoting systemic metabolic balance and reducing systemic inflammation levels. Exercise-induced physiological adaptations involve complex molecular pathways. For instance, studies reveal that mitochondrial open reading frame 12S rRNA-c (MOTS-c) and aerobic training activate the NRG1-ErbB4-C/EBPβ pathway to induce cardiac physiological adaptations, improving myocardial morphology and function (36). This enhancement of cardiac function contributes to elevated overall physiological reserve, counteracting fatigue. More importantly, aerobic exercise effectively modulates inflammatory responses. A cross-sectional study indicated that habitual aerobic exercise correlates with lower levels of inflammatory markers, such as white blood cell, neutrophil, and platelet counts, with this anti-inflammatory effect partially mediated by elevated total serum bilirubin levels—a substance possessing antioxidant and anti-inflammatory properties (37). Long-term voluntary aerobic exercise in mice demonstrated that exercise is closely linked to maintaining physiological function during aging by altering plasma metabolomics, particularly through increased levels of metabolites like spermidine associated with cellular quality control (38). Enhanced autophagy facilitates the clearance of damaged cellular components and maintains intracellular homeostasis, which is crucial for alleviating chronic fatigue and stress. Furthermore, studies on high-intensity interval training (HIIT) in patients with metabolic syndrome demonstrate that exercise improves metabolic syndrome scores, with this improvement particularly correlated with reductions in blood pressure and waist circumference (39). The amelioration of metabolic syndrome signifies enhanced insulin sensitivity, optimized lipid metabolism, and decreased inflammatory markers. These systemic beneficial changes collectively create a favorable physiological internal environment conducive to alleviating psychological distress and improving sleep.

### 3.2 Clinical evidence and intervention outcomes

Aerobic exercise demonstrates clear clinical evidence in improving sleep quality among elderly breast cancer patients. A 16-week pilot study examined the effects of moderate-to-high-intensity aerobic exercise combined with resistance training, delivered in a circuit-based, interval pattern, on sleep quality among cancer survivors (9). The study randomly assigned sedentary, overweight, or obese survivors of breast, prostate, and colorectal cancers to either an exercise group (n=60) or a usual care group (n=30). The exercise intervention comprised three weekly sessions of supervised, moderate-to-high-intensity aerobic exercise (65-85% VO2max) and resistance training (65-85% 1RM) performed in a circuit-interval format. Sleep quality was assessed using the Pittsburgh Sleep Quality Index (PSQI). Results showed significant improvement in PSQI total scores in the exercise group compared to the usual care group post-intervention (mean difference between groups: -2.7; 95% CI, -4.2 to -0.6), with lower PSQI scores indicating improved subjective sleep quality (8). Further analysis revealed a significant negative correlation between PSQI improvement and reduced insulin resistance (assessed via HOMA-IR) in the exercise group (r = -0.91; p<0.01), suggesting that exercise-induced sleep quality improvement may be associated with positive changes in metabolic health (8). This study provides strong evidence that aerobic combined with resistance training serves as an effective non-pharmacological intervention to improve sleep quality in cancer survivors, including breast cancer patients, and may confer metabolic benefits.

Even during exceptional periods like the COVID-19 pandemic, aerobic exercise interventions demonstrated adaptability and efficacy. An exploratory study evaluated the impact of a 16-week online home-based exercise program on sleep quality in breast cancer patients (10). The program delivered functional training via Zoom, with 15 participants completing the intervention. Sleep quality was assessed using the PSQI and accelerometers. Results showed a significant decrease in participants’ PSQI total scores, indicating improved subjective sleep quality manifested as shorter sleep latency, increased total sleep time, and enhanced sleep efficiency. Objective measurements further supported these findings, demonstrating increased total sleep time and reduced average wake duration. This pilot study demonstrates that online aerobic exercise interventions can effectively enhance sleep quality among breast cancer survivors. It underscores the importance of integrating exercise into patient care, particularly during exceptional circumstances like pandemic lockdowns, offering a viable alternative for patients unable to participate in in-person group exercise sessions (10).

### 3.3 The efficacy of aerobic exercise in alleviating psychological distress

As a non-pharmacological intervention, aerobic exercise demonstrates significant potential in alleviating psychological distress among cancer patients, particularly elderly breast cancer patients. Its core mechanism lies in enhancing overall mental health by improving emotional regulation and reducing anxiety and depressive symptoms. Multiple studies confirm that aerobic exercise effectively regulates neurotransmitter and hormone levels, thereby improving emotional states (40). For instance, a one-year randomized controlled trial involving prostate cancer patients found that those receiving supervised aerobic and resistance combined training exhibited significantly reduced depression and anxiety symptoms at the midpoint of the intervention (6 months) (41). Similarly, studies in fibromyalgia patients indicate that aerobic exercise improves depression, anxiety, and stress levels through its neuroendocrine system remodeling effects (40). A systematic review and meta-analysis for lung cancer patients further supports this conclusion, showing that exercise interventions, particularly those incorporating aerobic exercise, significantly reduce anxiety and depression levels (42).Collectively, this evidence indicates that aerobic exercise effectively mitigates negative emotional experiences through direct physiological mechanisms, such as promoting endorphin release and regulating hypothalamic-pituitary-adrenal axis function, thereby providing emotional buffering for patients.

Beyond direct psychophysiological benefits, aerobic exercise interventions indirectly alleviate psychological stress by fostering social interaction and enhancing self-efficacy. Participation in structured, often group-based exercise programs creates valuable social opportunities, helping reduce loneliness and build social support networks (43). A pilot study among patients receiving opioid agonist therapy found that despite low intervention attendance, participants reported that exercising in a clinic setting fostered connections with clinicians and enhanced social engagement and self-confidence. This sense of social connection and support from peers and professionals serves as a potent psychological resource. Simultaneously, successfully achieving exercise goals, such as maintaining training consistency or improving physical fitness,significantly boosts patients’ self-efficacy, or their belief in their ability to overcome challenges and accomplish tasks. This heightened sense of control is crucial for individuals enduring long-term illness. For instance, studies among breast cancer patients indicate that exercise interventions, by improving physical function and self-management skills, help alleviate treatment-related helplessness and psychological burdens (44). Thus, aerobic exercise not only directly influences mood but also multidimensionally alleviates patients’ psychological distress by fostering a positive psychosocial environment and a sense of personal capability.

## 4 Effects of mind-body exercises on sleep and mental health in elderly breast cancer patients

### 4.1 Theoretical basis of mind-body exercises

Mind-body exercises (MBEs) constitute a comprehensive intervention integrating physical movements, breath regulation, and mental focus. Their theoretical foundation is rooted in autonomic nervous system regulation and psychophysiological integration mechanisms. These practices encompass diverse forms among yoga, tai chi, qigong, and meditation. Through specific postures, breathing rhythms, and guided attention, they regulate sympathetic nervous system excitability, promoting psychological relaxation and physiological equilibrium (45). Research indicates that mind-body exercises significantly reduce cortisol levels and increase parasympathetic activity, thereby improving stress responses and emotional states (46). This effect is particularly pronounced in older adults, where sustained practice enhances autonomic nervous system adaptability and reduces age-related increases in sympathetic nervous system tension (47).

From a cognitive-emotional regulation perspective, mind-body practices exert effects through dual pathways: On one hand, repetitive movement patterns and breathing rhythms can form conditioned responses, inhibiting excessive amygdala activation and reducing emotional reactivity to negative stimuli (48). On the other hand, non-judgmental awareness of present experiences can reshape the prefrontal cortex’s regulatory function over the limbic system, enhancing emotional regulation capacity (49).Randomized controlled trials show that breast cancer survivors undergoing 12 weeks of yoga intervention exhibit significantly reduced negative emotion scores. Functional magnetic resonance imaging (fMRI) reveals enhanced default mode network connectivity, closely associated with reduced self-referential thinking and improved emotional stability (50).

At the neuroplasticity level, mind-body practices promote brain-derived neurotrophic factor (BDNF) secretion, strengthening synaptic connections between the hippocampus and prefrontal cortex to improve cognitive function and psychological resilience (51). A network meta-analysis of Parkinson’s disease patients found that yoga practitioners significantly outperformed conventional exercise groups in Unified Parkinson’s Disease Rating Scale (UPDRS) motor scores and depression symptom improvement, suggesting mind-body practices possess unique neuroprotective effects against neurodegenerative diseases (52). Furthermore, through functional reorganization of the somatosensory cortex and insula, these practices enhance perceptual accuracy of internal physiological signals, a change positively correlated with improved emotional regulation capacity (53).

From a traditional medical perspective, mind-body practices like qigong and tai chi originate from Chinese medical theory of unity of body and spirit, emphasizing the restoration of yin-yang balance through the coordinated regulation of posture, breathing, and mind (54). Modern research confirms that this holistic view aligns closely with contemporary psychosomatic medicine theories of multi-system interactions, such as the brain-gut axis, and brain-immune axis. For instance, practicing baduanjin significantly improves circadian rhythm stability in sub-health individuals while regulating yang deficiency and yin deficiency constitution scores, indicating its bidirectional regulation of autonomic nervous function and metabolic status (55). This multi-targeted, holistic action makes mind-body practices particularly suitable for addressing the common comorbidity of sleep disorders and psychological distress in elderly breast cancer patients. Aerobic exercise and mind-body practices exert differing effects on sleep quality through distinct pathways, as shown in **Table 1**.

**Table 1:** Characteristics of included studies on exercise and mind-body interventions for sleep quality and psychological outcomes in cancer survivors.

Intervention	Authors	Sleep quality metrics	Psychological distress indicators	Physiological indicators	Key findings
AE	Normann et al. (2022) (9)	PSQI	NR	insulin resistance	Improvements in PSQI are significantly associated with reduced insulin resistance
AE	Sagarra-Romero et al. (2022) (10)	Shorter sleep latency Increased total sleep time Improved sleep efficiency	NR	NR	improvements in subjective and objective sleep quality.
AE	Talotta et al. (2022) (40)	PSQI	Depression, anxiety, and stress levels	Rewiring of the Neuroendocrine System	Exercise improves depression, anxiety, and stress by regulating neurotransmitters and hormones
AE	Galvao et al. (2020) (41)	NR	Depression and anxiety	Muscle Strength Functional Capacity Hormones	Combined aerobic and resistance training significantly reduces psychological distress
AE	De Nys et al. (2024) (68)	PSQI	psychological distress	Decreased cortisol levels	Physical activity can slightly reduce cortisol levels and improve sleep quality
Mind-body Practice	Gregoire et al. (2020) (11)	MFI-20 Fatigue Inventory, ISI Insomnia Severity Index, Wrist-Worn Sleep Monitor	HADS Anxiety and Depression Scale PSWQ Worry Questionnaire	Wrist-based activity tracker monitors steps and hours of physical activity	Self-care interventions significantly improved fatigue, sleep, emotional distress, and cognitive function
MBP	Van der et al. (2020) (73)	NR	Depression Anxiety Stress Scale Emotional Distress	Functional brain connectivity Structural brain changes	Mindfulness Intervention Alleviates Emotional Distress and Fatigue
MBP	Osypiuk et al. (2020) (78)	Symptoms of Sleep Disorders	Depression Anxiety Stress	Pain Fatigue Nociceptive Awareness	Fatigue, anxiety, depression, perceived stress, and self-esteem, significant improvements were demonstrated
MBP	Wong et al. (2022) (63)	Polysomnography	State-Trait Anxiety Inventory, Pre-Sleep Arousal Scale	Sleep architecture sleep fragmentation index, slow-wave activity spectral band power	Subjective sleep quality improved across the board
MBP	Liu et al. (2022) (66)	PSQI Pittsburgh Sleep Quality	Anxiety, depression	Grip Strength, Joint Range of Motion, Lung Capacity	Yoga Improves Sleep Quality
MBP	Liu et al. (2023) (69)	PSQI Pittsburgh Sleep Quality Index,VSHSS Sleep Scale	NR	NR	Taichi significantly improves sleep quality
MBP	Wunderlin et al. (2024) (62)	PSQI Pittsburgh Sleep Quality Index, Polysomnography, Subjective Sleep Quality	FFMQ-SF Five-Factor Mindfulness Questionnaire	High-frequency power low-wave activity	Individuals with high mindfulness report better subjective sleep quality

AE, Aerobic Exercise; MBP: Mind-body practice; PSQI, Pittsburgh Sleep Quality Index; NR, Not Reported; HADS, Hospital Anxiety and Depression Scale; MFI-20, Multidimensional Fatigue Inventory-20; VSHSS, Verran and Snyder-Halpern Sleep Scale; FFMQ-SF, Five-Facet Mindfulness Questionnaire-Short Form; STAI, State-Trait Anxiety Inventory; PSAS, Pre-Sleep Arousal Scale; PSWQ, Penn State Worry Questionnaire.

### 4.2 Clinical research findings

Clinical studies have identified diverse intervention pathways to improve sleep quality and psychological distress among cancer patients, particularly elderly breast cancer patients. A randomized controlled trial involving cancer patients who had completed treatment evaluated the efficacy of a group intervention combining self-care with self-hypnosis (11). This study enrolled 95 female patients with various cancers. Results demonstrated significant group-by-time interaction effects in the intervention group compared to the waitlist control group, indicating marked improvements in fatigue, sleep, emotional distress, and cognitive function. This study confirmed that group interventions integrating self-hypnosis techniques effectively enhance post-treatment quality of life for cancer patients, particularly in alleviating fatigue, improving sleep, and reducing emotional distress (56). This demonstrates that interventions centered on psychological skill training can directly influence patients’ psychophysiological processes, yielding multidimensional health benefits.

Further research revealed a strong association between intervention outcomes and specific psychological factors. In the aforementioned study combining self-care with self-hypnosis, researchers explored predictors of fatigue symptom evolution (57, 58). Analysis indicated that depression, anxiety, and worry were key factors predicting fatigue changes. This implies that patients’ levels of emotional distress, particularly depression, anxiety, and disease-related worry, directly influence their response to interventions and the trajectory of symptom improvement, such as fatigue. This finding underscores the necessity of fully considering and actively addressing patients’ psychological distress when designing and implementing intervention programs. Improving emotional states may serve as a crucial pathway to effectively reduce fatigue and thereby comprehensively enhance quality of life (11, 59). Consequently, interventions targeting psychological factors constitute a core component for enhancing overall therapeutic efficacy.

### 4.3 The integrated mechanism of mind-body practices on sleep and mental health

Mind-body practices such as mindfulness, meditation, yoga, tai chi, and qigong demonstrate a unique multidimensional mechanism for improving sleep quality and psychological distress in elderly breast cancer patients by integrating psychological regulation with behavioral modification. Their core lies in regulating emotional and cognitive processes by enhancing nonjudgmental awareness of present experiences, thereby indirectly optimizing sleep structure and rhythms. Research indicates that individuals with higher trait mindfulness levels often report better subjective sleep quality (60). This association may stem from mindfulness-facilitated emotional regulation. Specifically, mindfulness disrupts the vicious cycle linking stress to sleep disturbances by reducing rumination, repetitively dwelling on negative emotions in a passive manner (61). A study of healthcare workers found that both trait and state mindfulness promote sleep health by decreasing rumination and negative affect, with benefits primarily manifesting in subjective sleep dimensions.

This suggests that mind-body practices first facilitate psychological decoupling from daytime emotional stress, creating a tranquil mental environment conducive to nighttime sleep.

At the neurophysiological level, the sleep-improving effects of mind-body practices may manifest in specific EEG characteristics. Research indicates that higher trait mindfulness correlates positively with the percentage of rapid eye movement (REM) sleep stages and is associated with lower high-frequency EEG activity during both REM and non-REM (NREM) periods (62). REM sleep is crucial for emotional processing, while high-frequency activity during NREM sleep is often regarded as a marker of cognitive hyperarousal. Thus, mind-body practices may promote optimal emotional regulation by enhancing REM sleep function while suppressing NREM hyperarousal, thereby consolidating sleep and alleviating psychological distress through neural mechanisms (62). Furthermore, studies comparing mindfulness therapy with conventional sleep hygiene education reveal differing effects on sleep macrostructure despite both improving self-reported sleep quality: Mindfulness intervention significantly increased N2 stage sleep, whereas sleep hygiene education increased N3 stage sleep (63). This suggests mindfulness may improve sleep through mechanisms distinct from traditional behavioral interventions, focusing more on influencing specific sleep stages via psychological regulation.

**Figure 2:**
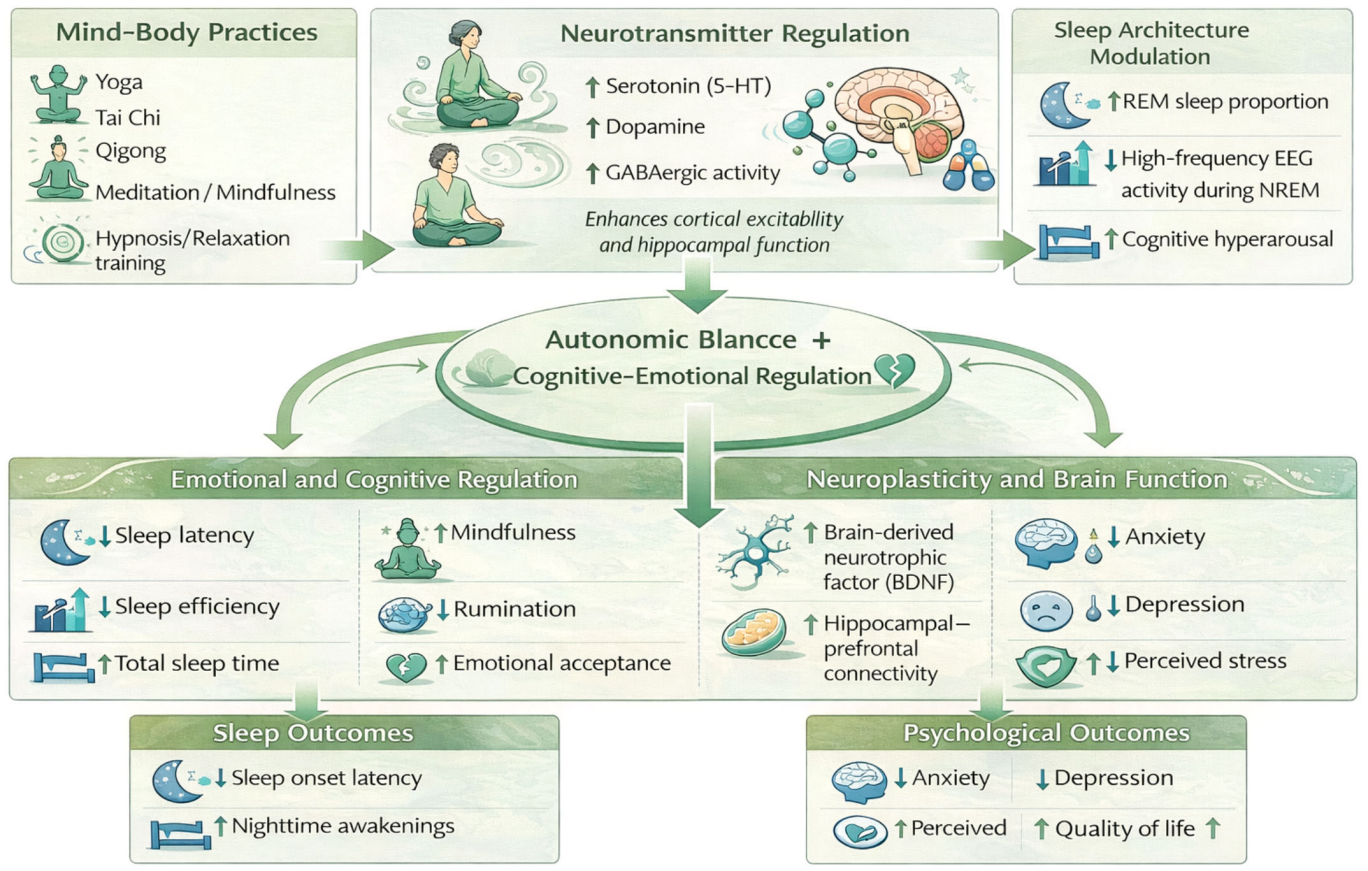
Mechanisms by which mind–body practices improve sleep quality and psychological health in older breast cancer patients.

Beyond direct sleep regulation, mind-body practices comprehensively improve patients’ physical and mental states by promoting cognitive recovery and enhancing overall quality of life. Mindfulness training enhances individual focus and reduces task-irrelevant mind wandering (64). Sleep distress has been identified as a trigger for mind wandering, and mind-body practices may reduce this cognitive interference by improving sleep (64). Such cognitive enhancement is particularly crucial for cancer patients. A pre-rehabilitation study involving breast cancer surgery patients found that those receiving mind-body pre-rehabilitation interventions demonstrated significantly greater cognitive improvements compared to the exercise pre-rehabilitation group (65). Concurrently, mind-body practices effectively reduce anxiety, depression, and stress levels (65), potentially through enhanced psychological resilience. Research confirms that psychological resilience mediates the relationship between mindfulness levels and sleep quality (66). Through practice, patients cultivate the ability to approach disease-related stress and physical discomfort with acceptance and non-judgment, thereby strengthening their psychological resilience. This resilience buffers the negative impact of stress on sleep and mood, ultimately enhancing their quality of life (67). This chain reaction from psychological regulation to behavioral adaptation to physiological improvement, constitutes the core mechanism underlying the comprehensive therapeutic effects of mind-body exercises on sleep and psychological distress in elderly breast cancer patients, as summarized in **Figure 2**.

## 5 Comparison of therapeutic effects between aerobic exercise and mind-body practices

### 5.1 Comparison of effects on sleep quality

**Figure 3:**
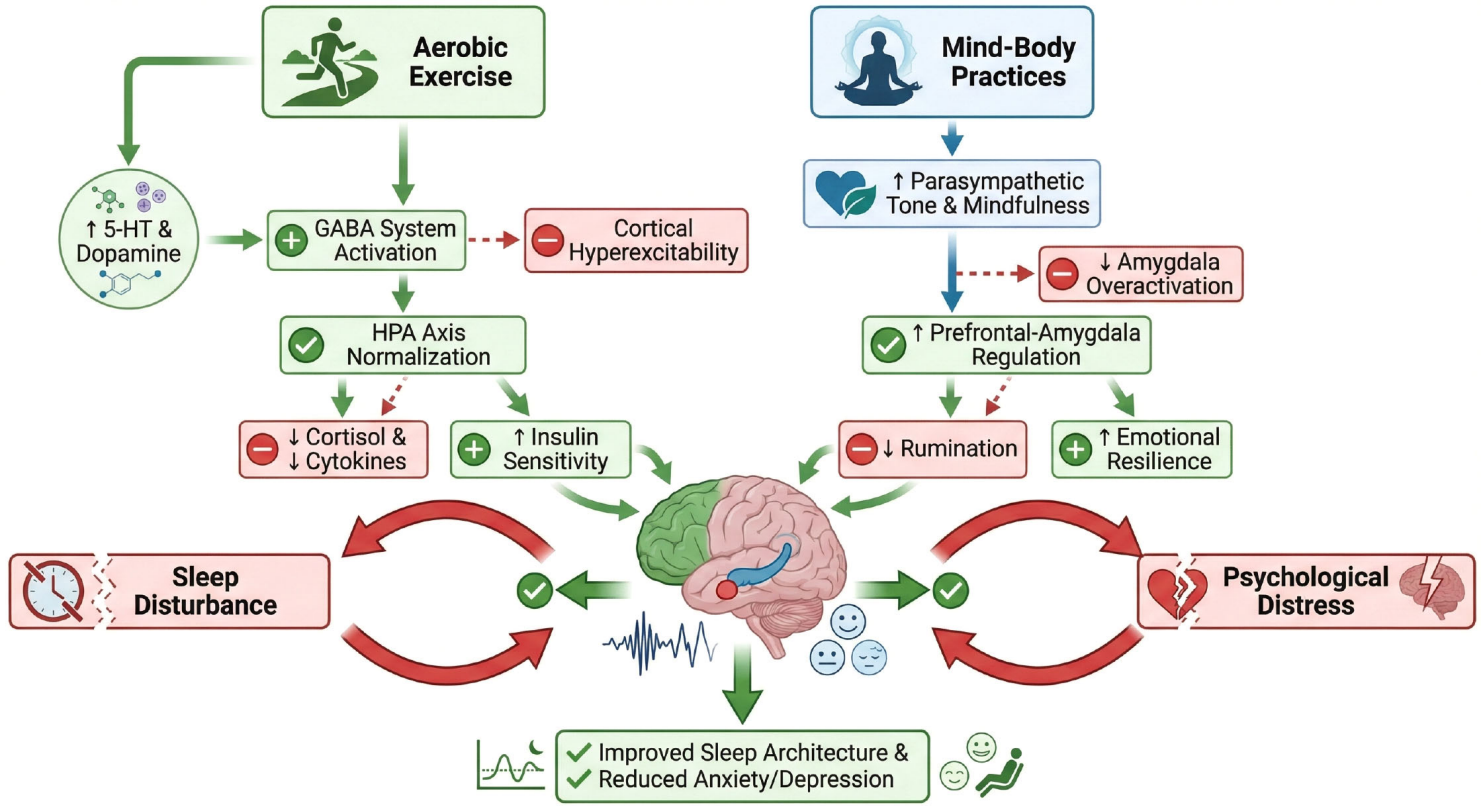
Comparative mechanisms of aerobic exercise versus mind–body practices on sleep and psychological outcomes in older breast cancer patients.

A comparative overview of the distinct mechanisms by which aerobic exercise and mind-body practices exert their effects on sleep and psychological outcomes is presented in **Figure 3**. Aerobic exercise demonstrates significant efficacy in improving sleep quality among elderly breast cancer patients, particularly in enhancing sleep architecture and physiological indicators. Multiple studies indicate that aerobic exercise effectively increases sleep efficiency (SE) and total sleep time (TST) while reducing sleep latency (SL). For instance, a randomized controlled trial involving breast cancer patients found that a 16-week aerobic and resistance exercise intervention significantly improved Pittsburgh Sleep Quality Index (PSQI) scores, with this improvement significantly correlated with reduced insulin resistance (8). Additionally, another study demonstrated that aerobic exercise further optimizes sleep quality by regulating cortisol levels and enhancing autonomic nervous system function (68). These physiological improvements were not only statistically significant but also demonstrated high sustainability in clinical practice, providing a reliable non-pharmacological intervention for long-term sleep management in elderly breast cancer patients.

In contrast, mind-body practices show greater efficacy in improving sleep latency and sleep maintenance, particularly benefiting patients with psychological distress. A study on yoga-based rehabilitation training for breast cancer patients found that a 12-week intervention significantly reduced PSQI scores and improved sleep quality through breathing regulation and relaxation techniques (69). Another systematic review on traditional Chinese exercises also indicated that such interventions significantly enhance sleep quality in specific populations, particularly by optimizing sleep architecture while alleviating anxiety and depressive symptoms (70). Notably, the advantage of mind-body practices lies in their multimodal intervention characteristics, addressing both psychological and physiological dimensions of sleep disorders. This offers a more comprehensive treatment approach for elderly breast cancer patients experiencing psychological distress.

In summary, aerobic exercise and mind-body practices each have distinct strengths in improving sleep quality. Aerobic exercise is more suitable for patients requiring significant improvements in sleep architecture and physiological indicators, while mind-body practices are better suited for those experiencing psychological distress or difficulty maintaining sleep. In clinical practice, personalized intervention plans can be selected based on patients’ specific symptoms and preferences.

### 5.2 Comparison of efficacy in alleviating psychological distress

Mind-body exercises demonstrate unique advantages in improving psychological distress among elderly breast cancer patients. Multiple studies indicate that such interventions, through targeted emotion regulation techniques and cognitive-behavioral adjustment strategies, can significantly reduce patients’ depressive and anxiety symptoms (71). Long-term follow-up data from the OptiTrain trial revealed that patients who participated in mind-body exercises maintained lower emotional symptom burden 12 months after chemotherapy completion. Improvements were particularly pronounced in core symptoms such as feeling sad (ES = -0.44) and irritability (ES = -0.44). This sustained psychological benefit may stem from mind-body exercises fostering patients’ cognitive restructuring abilities, enabling them to better cope with disease and treatment-related stress. An online intervention study (OPaCT) targeting breast cancer patients during chemotherapy further confirmed that mind-body interventions combined with psychological support significantly improved psychological needs scores and reduced unmet psychological care needs (72). Additionally, mindfulness interventions have been shown to effectively alleviate psychological distress in breast cancer patients by enhancing emotional regulation and reducing rumination (73). This intervention is particularly suitable for elderly patients due to its moderate intensity and emphasis on self-acceptance, which aids in establishing long-term psychological adaptation mechanisms.

Aerobic exercise exerts comprehensive stress-relieving effects through dual physiological and psychological pathways. Research indicates that regular aerobic exercise modulates hypothalamic-pituitary-adrenal axis function and lowers cortisol levels, thereby reducing stress responses (9). Concurrently, exercise-induced endorphin release generates natural euphoria and improves emotional states. In the OptiTrain trial, patients in the aerobic exercise group demonstrated significant improvements in emotional symptoms during chemotherapy, including reductions in feeling sad (ES = -0.56) and irritability (ES = -0.31) (71). Notably, the efficacy of aerobic exercise in alleviating psychological distress correlates with exercise intensity: with moderate-intensity (65-85% VO_2_max) interval training demonstrating optimal mood regulation effects (9). Furthermore, group exercise formats provide social support, further enhancing psychological benefits. The Neo-train trial found that breast cancer patients engaging in aerobic exercise not only experienced reduced psychological distress but also significantly improved treatment tolerance, manifested as higher relative dose intensity and shorter hospital stays (74). This characteristic of comprehensive mind-body improvement positions aerobic exercise as a multifaceted intervention for alleviating psychological distress.

In comparison, mind-body practices are more targeted toward emotional regulation and cognitive restructuring, particularly suited for patients with pronounced cognitive distortions or emotional regulation difficulties (73). Aerobic exercise, however, produces broader stress-relieving effects through diverse mechanisms such as neuroendocrine regulation and social interaction (9, 71). Clinical selection should consider individual patient characteristics, including physical functional status, psychological symptom profiles, and preferences. When necessary, combining both approaches may yield synergistic effects.

### 5.3 Adherence and safety analysis of interventions

Aerobic exercise demonstrated good adherence among elderly breast cancer patients; however, its high physical demands may limit participation for some older adults. Multiple studies indicate that aerobic exercise interventions typically require patients to possess a certain baseline level of physical fitness, including cardiorespiratory endurance and muscular strength, which may pose challenges for elderly patients—particularly those with comorbid chronic conditions (75). For instance, the high-intensity aerobic exercise protocol in the ATOPE trial, while demonstrating preventive effects against cardiac toxicity, required patients to complete 12–18 training sessions prior to treatment, a commitment that may be difficult for frail elderly patients to sustain (75).

Adherence is influenced by multiple factors including physical fitness levels, facility accessibility, and supervision models. Regarding facilities, the requirement to travel to dedicated locations for training may increase patients’ transportation burden and time costs, posing barriers to consistent participation. Concerning supervision models, the EXERT-BC study indicates that while dose-escalating resistance training protocols demonstrate good safety in breast cancer survivors, the execution of high-intensity compound movements necessitates professional supervision, potentially limiting their implementation in community settings (76). Notably, remote supervision models may mitigate this issue—online resistance training delivered during the COVID-19 pandemic demonstrated attendance rates of 86%–91%, significantly higher than the 80%–82% observed in in-person classes (77), suggesting technology-assisted approaches could enhance accessibility and adherence among elderly patients.

Adherence is influenced by multiple factors including physical fitness levels, facility accessibility, and supervision models. Regarding facilities, the requirement to travel to dedicated locations for training may increase patients’ transportation burden and time costs, posing barriers to consistent participation. Concerning supervision models, the EXERT-BC study indicates that while dose-escalating resistance training protocols demonstrate good safety in breast cancer survivors, the execution of high-intensity compound movements necessitates professional supervision, potentially limiting their implementation in community settings (76). Notably, remote supervision models may mitigate this issue—online resistance training delivered during the COVID-19 pandemic demonstrated attendance rates of 86%–91%, significantly higher than the 80%–82% observed in in-person classes (77), suggesting technology-assisted approaches could enhance accessibility and adherence among elderly patients.

Regarding safety, aerobic exercise is generally safe under standardized guidance and appropriate supervision, though potential risks warrant attention. Older adults may experience increased risks of musculoskeletal injuries, cardiovascular events, or falls due to inappropriate exercise intensity, improper form, or poorly managed underlying conditions. Therefore, comprehensive pre-exercise assessments, professional supervision during sessions, and post-exercise monitoring of outcomes are critical for ensuring safety.

In contrast, mind-body practices offer greater safety and convenience for home implementation. The QMBE (Qigong Mind-Body Exercise) study found no serious adverse events reported among eighteen breast cancer survivors after a 12-week intervention, with 80% maintaining high adherence (78). This low-intensity, self-regulated nature makes it particularly suitable for elderly patients with physical limitations, in terms of adherence, the home-based feasibility of mind-body exercises is a significant advantage. In the Guolin Qigong trial, patients achieved significant improvements in fatigue and sleep quality by attending weekly group sessions combined with home practice, with 91% completing follow-up assessments (79). Furthermore, the MAMA_MOVE Gaia project found that group mind-body exercise classes were not only highly safe but also stabilized patients’ post-treatment quality of life scores, with 54% of participants attending over 70% of sessions (80). This combination of social support and low physical exertion may be key factors explaining why mind-body exercises demonstrate superior adherence compared to aerobic exercise in older adults (81). Notably, the Fit2ThriveMB program for metastatic breast cancer patients further demonstrated that personalized mind-body interventions using mobile health technology, which combined Fitbit monitoring and weekly guidance, achieved a 92.7% device wear rate and 53.1% daily step goal attainment rate, with no related adverse events (82). This offers novel insights for home-based rehabilitation.

In terms of safety, mind-body exercises rarely cause serious adverse events due to their low-intensity, low-impact nature. Common side effects are typically mild muscle soreness or fatigue, which usually resolve spontaneously with rest. For elderly patients with osteoporosis, joint disorders, or balance impairments, modified forms such as seated Tai Chi or simplified yoga can further reduce risks.

Based on the above analysis, we propose a three-dimensional feasibility-safety-effectiveness evaluation framework for elderly breast cancer patients to guide the selection and individualized adjustment of intervention programs in clinical practice.

The feasibility dimension assesses the acceptability and accessibility of intervention programs within the target population, whether special equipment or facilities are required, whether implementation is feasible in a home environment, whether professional supervision is necessary, whether physical limitations of elderly patients are considered, and whether social support is provided. Within this dimension, mind-body exercises typically outperform aerobic exercise, particularly for elderly patients with mobility limitations or transportation challenges.

The safety dimension evaluates the risk level of intervention programs in the elderly population, including risk of exercise-related injuries, risk of cardiovascular events, risk of falls, and incidence of adverse events. In this dimension, mind-body exercises demonstrate clear advantages due to their low-intensity, low-impact nature, making them especially suitable for elderly patients with multiple comorbidities or poor functional status.

The effectiveness dimension evaluates the intervention’s impact on target symptoms, including: improvement in sleep quality, reduction in psychological distress, changes in physiological indicators, and enhancement of functional capacity. In this dimension, aerobic exercise excels at improving sleep architecture and metabolic markers, while mind-body practices demonstrate greater efficacy in enhancing subjective sleep perception and emotional regulation.

In clinical practice, a balanced approach among the three modalities should be tailored to individual patient characteristics. For patients with good physical fitness primarily needing sleep structure and metabolic state improvements, aerobic exercise may be prioritized, though feasibility and safety must be carefully considered. For those with limited physical capacity primarily requiring improvements in subjective sleep perception and psychological distress, mind-body exercises should be prioritized due to their clear advantages in feasibility and safety. For patients with complex functional states, a combined intervention strategy may be employed, starting with mind-body exercises and gradually introducing aerobic exercise to achieve the optimal risk-benefit balance.

Based on existing evidence, we propose the following practical recommendations to improve intervention adherence among elderly breast cancer patients. First, adopt remote supervision models, such as video-conference guided group sessions, to reduce transportation burdens while maintaining professional guidance (77). Second, integrate mobile health technologies, including wearable devices and mobile applications, to provide real-time feedback and personalized coaching (82). Third, emphasize social support by enhancing patients’ sense of social connection and belonging through group sessions or peer support groups (80). Fourth, offer multiple intervention formats, allowing patients to select appropriate aerobic or mind-body exercises based on personal preferences and physical capacity. Finally, adopt a progressive intervention strategy, starting with low-intensity, short-duration exercises and gradually increasing intensity and duration to help patients build confidence and establish exercise habits.

### 5.4 Potential synergistic effects of combined interventions

Previous sections have elucidated the distinct yet complementary mechanisms by which aerobic exercise and mind-body practices improve sleep quality and psychological distress in elderly breast cancer patients. Aerobic exercise primarily acts on physiological pathways, including hypothalamic-pituitary-adrenal axis regulation, metabolic enhancement, and inflammation reduction (9, 37, 68). In contrast, mind-body practices primarily influence psychological and neurocognitive mechanisms, such as emotional regulation, mindfulness enhancement, and stress reduction (11, 62, 63). This complementary mechanism suggests that combining these two interventions may yield synergistic effects exceeding the sum of their individual contributions.

The physiological and psychological pathways targeted by aerobic exercise and mind-body practices are not mutually exclusive but interact in complex ways. Aerobic exercise-induced improvements in sleep architecture and metabolic function may create a more favorable physiological state, thereby enhancing the effects of mind-body practices. Conversely, the emotional regulation and stress reduction capabilities gained through mind-body practices may increase patients’ motivation, self-efficacy, and adherence to aerobic exercise. This bidirectional promotion suggests combined interventions may yield additive or even synergistic effects on clinical outcomes.

From a neurobiological perspective, aerobic exercise promotes neuroplasticity through mechanisms such as upregulation of brain-derived neurotrophic factor and increased cerebral blood flow (48), potentially establishing the neural foundation for mind-body practice’s cognitive and emotional training effects. Mind-body practice enhances the prefrontal cortex’s regulatory capacity over limbic system activity (49), potentially optimizing the neural environment for exercise-induced neuroplastic changes. The integration of these complementary mechanisms may yield more pronounced and enduring improvements in sleep quality and psychological distress.

Although direct evidence for combined aerobic exercise and mind-body practice interventions in elderly breast cancer patients remains limited, preliminary studies in relevant populations support the potential benefits of integrated approaches. Knoerl et al. (65) compared exercise-based prehabilitation with mind-body prehabilitation in women undergoing breast cancer surgery. Both interventions improved psychological outcomes, but the mind-body group demonstrated superior cognitive function enhancement. This suggests different interventions may target distinct outcome domains, providing theoretical support for combined approaches.

In the OptiTrain trial (71), patients receiving either resistance-HIIT or aerobic-HIIT demonstrated significantly reduced emotional symptom burden compared to standard care, with effects persisting at 12-month follow-up. While this trial did not explicitly test combined aerobic and mind-body exercise interventions, it demonstrated that different exercise modalities can produce complementary benefits across multiple symptom domains.

Research in non-cancer populations offers additional support. Studies involving individuals with metabolic syndrome indicate that combining aerobic exercise with stress reduction techniques yields greater improvements in metabolic parameters and mental health than either intervention alone (39). Similarly, among older adults with insomnia, a combined approach integrating physical activity and mindfulness techniques demonstrated superior sleep outcomes compared to single interventions (63).

Based on the underlying mechanisms and preliminary evidence, we propose the following models integrating aerobic exercise with mind-body practices for exploration in future research. Sequential Intervention Model: In this approach, patients first undergo one intervention followed by another, either in a fixed sequence or adjusted according to individual characteristics. For instance, patients with severe anxiety may begin with mind-body practice to enhance emotional regulation and stress management, gradually introducing aerobic exercise once psychological stability is achieved. Conversely, patients primarily presenting with metabolic disorders may start with aerobic exercise, later incorporating mind-body practice to address residual psychological symptoms and support long-term adherence.

Synchronous intervention model delivers both interventions concurrently within the same timeframe. For instance, patients might attend two aerobic exercise sessions and two mind-body practice sessions weekly over 12–16 weeks. This approach maximizes synergistic potential but requires careful consideration of patient burden and scheduling feasibility.

In integrated Intervention model, elements of aerobic exercise and mind-body practice are integrated into a single session or program. Examples include mindfulness walking programs incorporating attention to breathing and bodily sensations during aerobic activity, or yoga interventions incorporating cardiovascular components. This integrated approach may enhance adherence by simplifying program logistics while potentially amplifying therapeutic effects by simultaneously engaging physiological and psychological mechanisms.

Stepwise intervention model matches intervention intensity and complexity to patient response, starting with simpler, lower-intensity interventions and progressively adding components as clinically warranted. For instance, patients might begin with a basic mind-body exercise program, with aerobic exercise added if sleep or psychological outcomes remain suboptimal after the initial phase. This personalized approach can optimize resource allocation while ensuring patients receive intervention intensity appropriate to their needs.

Future studies should systematically evaluate the efficacy, feasibility, and optimal implementation strategies of combined aerobic exercise and mind-body practice interventions in elderly breast cancer patients. Priority research questions are as follows:

Compared to single interventions, does combining aerobic exercise with mind-body practice yield superior improvements in sleep quality and psychological distress? This requires a three-arm randomized controlled trial comparing the combined intervention against each monotherapy and a control group. What is the optimal sequence and timing for combined interventions? Comparative effectiveness studies should evaluate the relative merits of sequential, concurrent, and integrated intervention models in terms of efficacy and adherence.Which patient subgroups benefit most from combined interventions? Moderator analyses should investigate whether baseline characteristics predict differential responses to combined versus single interventions. What mechanisms underlie synergistic effects? Mediator analyses should explore whether combined interventions yield benefits through additive or interactive effects across physiological and psychological pathways. What is the optimal “dose” for each intervention component in combined approaches? Dose-finding studies should determine the minimum effective dose of aerobic exercise and mind-body practice required to achieve synergistic effects while minimizing patient burden.

How can combined interventions be effectively implemented in clinical practice? Implementation science research should evaluate strategies for integrating aerobic exercise-mind-body practice programs into routine oncology care, including digital delivery platforms, community-based programs, and step-up care models.

Several practical issues warrant attention when developing combined interventions for elderly breast cancer patients. First, patient burden must be carefully managed, as combined interventions require greater time commitment than single interventions. Strategies to reduce burden include offering flexible scheduling, home-based options, and digital delivery platforms. Second, intervention sequencing should consider patient preferences and baseline capabilities, some patients may prefer starting with milder mind-body exercises before progressing to aerobic exercise. Third, supervision requirements may vary across interventions, with aerobic exercise typically necessitating more intensive safety monitoring, particularly in patients with lower fitness levels or frailty. Fourth, outcome assessments should capture both intervention-specific effects and shared outcomes to comprehensively describe intervention benefits.

In summary, the complementary mechanisms between aerobic exercise and mind-body practices provide a robust theoretical foundation for exploring combined interventions among elderly breast cancer patients experiencing sleep disturbances and psychological distress. Although direct evidence remains limited, preliminary studies in relevant populations support the potential for synergistic effects. Future research should employ rigorous trial designs to systematically evaluate combined intervention approaches, focusing on optimal sequencing, dosage, and implementation strategies. Such studies hold the potential to establish more effective, personalized rehabilitation protocols that fully leverage the full potential of non-pharmacological interventions available to this vulnerable population.

## 6 Limitations of existing research

### 6.1 General research design and sample limitations

Current studies investigating the effects of aerobic exercise and mind-body practices on improving sleep quality and psychological distress in elderly breast cancer patients exhibit significant limitations in research design and sample selection. First, most studies feature limited sample sizes and lack designs specifically tailored to elderly breast cancer patient populations. For example, a study investigating exercise adherence among breast cancer patients undergoing aerobic and resistance training during or after neoadjuvant chemotherapy included only 68 participants with an average age of 52 years, failing to specifically focus on the elderly population (79). This indicates existing research has insufficient recruitment and inclusion of adequate numbers of elderly breast cancer patients, limiting the representativeness and generalizability of findings. Small sample sizes make it difficult to detect subtle yet clinically significant effects of interventions, particularly in the highly heterogeneous elderly population, whose physiological function, comorbidities, and treatment responses may differ from younger patients. Therefore, future research requires larger-scale clinical trials specifically designed for elderly breast cancer patients to provide more targeted evidence.

Second, the number of high-quality randomized controlled trials remains insufficient, and existing studies exhibit significant variations in intervention duration and intensity, posing challenges for comparing efficacy and drawing definitive conclusions. For example, a mixed-methods randomized controlled trial exploring the effects of Guolin Qigong on cancer-related fatigue set its intervention cycle at 12 weeks with a 4-week follow-up period (75). However, intervention durations across studies range from weeks to months, and there is a lack of uniform standards for the frequency, single-session duration, and intensity of exercise or mind-body practices. This heterogeneity complicates data pooling and meta-analysis in systematic reviews, hindering the identification of optimal intervention dosages.

The heterogeneity of intervention protocols extends beyond duration, manifesting more prominently in insufficient standardization of frequency, intensity, intervention formats, and specific content descriptions. This heterogeneity poses multiple challenges for interpreting research findings and translating them into clinical practice. First, at the meta-analysis level, aerobic exercise intensity ranges widely across studies, single-session durations for mind-body practices vary from 30 to 90 minutes, and intervention cycles vary significantly from 4 to 24 weeks. These inconsistent parameters make direct comparisons between studies difficult, often resulting in high heterogeneity when pooling data and thereby reducing the reliability of meta-analysis conclusions. Second, at the clinical implementation level, the lack of standardized intervention protocols makes it challenging for clinicians to determine the most effective dose of intervention for elderly breast cancer patients and to accurately replicate the interventions used in studies. Notably, intervention descriptions are often overly brief, for instance, merely mentioning yoga training without detailing specific postures, breathing techniques, or meditation elements, or describing aerobic exercise without specifying exercise modalities. This severely limits the reproducibility of research.

### 6.2 Structured assessment of evidence quality

To further enhance the rigor of this systematic review, we conducted a structured assessment of the evidence quality in the included studies. Although no meta-analysis was conducted in this study, evidence quality was qualitatively assessed using the GRADE framework across five dimensions: risk of bias, inconsistency, indirectness, imprecision, and publication bias.

The Cochrane Risk of Bias tool (RoB 2.0) was used to assess risk of bias across included studies. Overall, study quality was moderate to high, but common issues included: First, due to the nature of exercise interventions, most studies could not blind participants, potentially introducing implementation bias. Second, some studies failed to explicitly report allocation concealment protocols, increasing the risk of selection bias. Third, a few studies had high loss-to-follow-up rates and did not perform intention-to-treat analyses, potentially affecting result reliability. However, most studies were well-controlled regarding outcome measurement blinding and selective reporting, resulting in an overall acceptable risk of bias.

Using the GRADE framework, we conducted a preliminary grading of evidence quality for primary outcome measures. Moderate-quality evidence supports aerobic exercise improving sleep quality. Although multiple randomized controlled trials consistently showed improved PSQI scores after aerobic exercise, such as Normann et al., most studies had methodological limitations due to lack of participant blinding and primarily enrolled non-elderly populations, and subjects were predominantly non-elderly populations. Thus, evidence quality was downgraded from high to moderate.

Low-quality evidence supports the efficacy of aerobic exercise in alleviating psychological distress. Studies by Galvao et al. (41) and Talotta et al. (40) indicate aerobic exercise reduces anxiety and depression levels. However, these studies involved prostate cancer patients and fibromyalgia patients, respectively, limiting their generalizability to elderly breast cancer patients. Additionally, some studies were narrative reviews rather than randomized controlled trials, resulting in low-quality evidence.

Moderate-quality evidence supports mind-body practices for improving sleep quality. Randomized controlled trials by Gregoire et al. (11) and Liu et al. (69), demonstrated significant improvements in PSQI scores following mind-body exercises, with moderate effect sizes. However, concerns exist regarding lack of participant blinding and high heterogeneity in intervention protocols. Nevertheless, consistent effect directions and adequate sample sizes led to a moderate-quality rating.

There is moderate-quality evidence supporting the efficacy of mind-body practices in alleviating psychological distress. Multiple RCTs demonstrated that improvements in anxiety and depression from mind-body practices persisted at 12-month follow-up, with moderate effect sizes. Despite some risk of bias and indirectness concerns, overall evidence quality was rated moderate.

Regarding comparisons of adherence and safety, only low-quality evidence currently exists. Comparative studies on intervention adherence and safety, Winters-Stone et al. (77) and Osypiuk et al. (78) were primarily observational or exploratory trials lacking randomized designs, limiting the strength of conclusions. Furthermore, high heterogeneity in outcome measures for adherence and safety across studies precluded quantitative synthesis, resulting in low evidence quality.

Overall, existing evidence supports the effectiveness of aerobic exercise and mind-body practices in improving sleep quality and alleviating psychological distress among elderly breast cancer patients. However, evidence quality is predominantly low to moderate, primarily constrained by indirectness of study populations, lack of blinding in intervention implementation, and heterogeneity of intervention protocols. Future research requires more high-quality randomized controlled trials specifically targeting elderly breast cancer patients, strictly adhering to the CONSORT statement and GRADE framework requirements to enhance the reliability and clinical applicability of the evidence.

### 6.3 Research gaps and future directions in aerobic exercise specificity

There is insufficient evidence to determine the optimal dose of aerobic exercise including frequency, intensity, duration, and intervention cycle for maximizing improvements in sleep and psychological symptoms among elderly breast cancer patients. Most studies employ arbitrarily set exercise parameters without systematically comparing the effects of different intensities or durations. Future studies should employ multi-arm randomized controlled trial designs to compare the effects of different exercise intensities and durations. This approach will establish clear dose-response curves, providing precise evidence for clinical prescribing.

Existing studies predominantly include relatively young breast cancer patients with good physical fitness, with insufficient attention to how subgroups exhibiting typical geriatric characteristics, such as frailty, sarcopenia, and multiple comorbidities adapt. Data on how elderly patients with varying baseline functional status adapt to aerobic exercise interventions remain scarce. Future studies should specifically recruit elderly breast cancer patients, incorporate geriatric-specific indicators such as frailty assessment, activities of daily living capacity, and fall risk, and explore strategies for developing individualized exercise prescriptions based on baseline functional stratification.

Most studies have short follow-up periods, lacking in-depth exploration of aerobic exercise long-term effects and behavioral maintenance mechanisms after intervention cessation. Research indicates that while most patients continue exercising after intervention, a significant proportion discontinue training immediately or within months. Discontinuation reasons include practical issues such as insufficient time and excessive distance to training venues. This suggests future studies should extend follow-up periods to 12 months or longer and employ mixed-methods approaches to explore psychosocial factors influencing long-term exercise adherence, such as self-efficacy, social support, and perceived barriers to exercise.

Future aerobic exercise-related studies should adopt more rigorous methodological designs. Specific recommendations are as follows: Conducting multicenter randomized controlled trials with a sample size of at least 200 participants to ensure sufficient statistical power for detecting clinically meaningful effects and supporting subgroup analyse. Employing objective sleep measurement tools, such as actigraphs and polysomnography, to complement subjective sleep questionnaires and provide a more comprehensive assessment of sleep architecture. Extending follow-up periods to at least 6 months, ideally reaching 12 months or longer, to evaluate the sustainability of intervention effects and identify factors influencing long-term adherence. Incorporating geriatric-specific outcome measures, including frailty indices, cognitive function assessments, physical performance tests, and quality-of-life scales tailored for older adults, to comprehensively capture intervention benefits in elderly patients. Implementing stratified randomization based on key patient characteristics to ensure balanced comparability between groups and facilitate more refined subgroup analyses.

### 6.4 Research gaps and future directions in the study of mind-body practice specificity

Mind-body practices such as yoga, tai chi, qigong, and mindfulness meditation exhibit significant differences in movement patterns, breathing techniques, and elements of mental focus. However, the current lack of head-to-head comparative research prevents determining which modality offers the greatest advantage for specific symptoms in elderly breast cancer patients, such as sleep disorders, anxiety, and depression. Future studies should conduct multi-arm randomized controlled trials to directly compare the intervention effects of different mind-body practice forms and explore their potential specific mechanisms of action.

Existing studies exhibit considerable variability in intervention frequency, session duration, and total intervention duration. Furthermore, descriptions of intervention content are often insufficiently detailed, limiting research reproducibility and clinical translation. Future studies should adhere to the TIDieR guidelines for detailed intervention reporting, explore the minimum effective dose—the lowest intervention intensity yielding clinically meaningful improvements, and develop modified mind-body practices suitable for elderly patients with mobility limitations.

Although existing studies suggest mind-body practices may exert effects through pathways such as autonomic nervous system regulation, psychological resilience enhancement, and sleep structure optimization, systematic mechanism research is lacking, particularly comprehensive evaluations involving neuroimaging, neuroendocrine, and immunological markers. Future studies should employ multimodal assessment tools, including functional MRI, cortisol circadian rhythm analysis, and inflammatory cytokine profiling, to elucidate the biological basis underlying mind-body exercises’ effects on improving sleep and psychological distress in elderly breast cancer patients.

Existing research on mind-body practices predominantly originates from Western contexts such as yoga and mindfulness interventions, with relatively limited studies on Eastern traditional practices like Tai Chi and Qigong, and a lack of cross-cultural comparisons. Future research should focus on mind-body practices grounded in Eastern cultural contexts, exploring their acceptability and efficacy among elderly breast cancer patients in local settings. Concurrently, the application value of digital platforms, such as mobile applications and remote live-streamed interventions, should be evaluated for promoting mind-body practices, particularly among elderly populations with mobility limitations.

To advance research in this field, future studies on mind-body practices should incorporate the following methodological recommendations. Designing multi-arm randomized controlled trials with a minimum sample size of 200 participants to enable direct comparisons between different mind-body practice modalities, and against active control groups. Employing objective sleep measurement tools, including actigraphs and polysomnography, to capture both subjective and objective dimensions of sleep quality and identify intervention-specific effects on sleep architecture.Establishing 6- to 12-month follow-up periods to assess intervention sustainability and identify factors associated with long-term practice maintenance, Incorporating age-specific outcome measures, such as cognitive function tests, physical performance assessments, frailty indices, and social engagement scales, to comprehensively capture the multidimensional benefits of mind-body practices in older adults, including mechanism-assessment indicators, including neuroimaging, stress and inflammation biomarkers, and psychological constructs to elucidate pathways of action, evaluate the feasibility and effectiveness of digital delivery platforms in enhancing accessibility and adherence among homebound or mobility-impaired elderly patients.

### 6.5 Summary of future research directions

In summary, future research requires designing larger-scale, methodologically rigorous clinical trials specifically targeting elderly breast cancer patients to provide more targeted evidence. Beyond addressing common methodological issues, researchers should prioritize intervention-specific research questions: establishing dose-response relationships for aerobic exercise while determining the relative efficacy of different mind-body practice modalities. For both intervention types, the following design elements should be prioritized, sample size ≥200 to ensure sufficient statistical power, incorporation of objective sleep measurement tools alongside subjective questionnaires, follow-up periods of at least 6–12 months to assess long-term effects, incorporating geriatric-specific outcome measures, conducting stratified randomization based on key patient characteristics, providing detailed intervention reporting following CONSORT and TIDieR guidelines. Additionally, exploring sociodemographic factors influencing long-term maintenance, such as age, education level, and marital status is crucial for enhancing intervention sustainability. Integrating digital health technologies may offer novel pathways to enhance intervention accessibility and adherence, particularly for mobility-impaired older adults. These research advances will propel the development of evidence-based precision rehabilitation programs, ultimately driving a paradigm shift from symptom management to holistic health promotion.

## 7 Perspectives

Rehabilitation management for elderly breast cancer patients presents a multidimensional, comprehensive clinical challenge. Sleep disorders and psychological distress, as common comorbid symptoms, significantly impair treatment adherence and quality of life. Existing evidence indicates that aerobic exercise and mind-body practices, as non-pharmacological interventions, demonstrate unique value and complementarity in improving health outcomes for this specific population.

At the mechanism level, aerobic exercise regulates hypothalamic-pituitary-adrenal axis function and promotes melatonin secretion, thereby improving sleep structure abnormalities and metabolic disorders. This physiological regulation is particularly crucial for elderly patients with metabolic syndrome. In contrast, mind-body practices activate the prefrontal cortex-limbic system neural circuitry to reduce sympathetic nervous system activity, thereby more effectively alleviating anxiety, depression, and chemotherapy-related cognitive impairment. This neuropsychological regulatory mechanism explains why mind-body practices excel in improving subjective sleep quality.

Given the significant heterogeneity in physiological function, psychological status, and comorbidities among elderly breast cancer patients, clinical practice should develop stratified, personalized intervention plans based on individual patient characteristics.

Stratified Recommendations Based on Physical Fitness, for patients with good physical fitness and strong aerobic capacity, moderate-intensity aerobic exercise is recommended as a priority. This includes brisk walking, stationary cycling, or swimming 3 times per week for 30–45 minutes per session. Such activities effectively improve cardiopulmonary function, metabolic status, and sleep architecture.

For individuals with poor physical fitness and limited aerobic capacity, a strategy combining low-intensity aerobic exercise with mind-body practices is recommended. Specifically, begin with low-intensity home walking, for instance, 3 sessions per week, 20 minutes per session, gradually transitioning to seated Tai Chi, simplified yoga, or mindful breathing exercises. This combined approach mitigates exercise-related risks while improving sleep and mental well-being through mind-body regulation mechanisms.

Tiered Recommendations Based on Psychological Distress Characteristics, for individuals primarily experiencing anxiety or worry, mind-body practices are prioritized, particularly mindfulness-based stress reduction, yoga, or tai chi. These interventions significantly alleviate anxiety symptoms by enhancing emotional regulation and reducing rumination. Recommend attending 2–3 group sessions weekly, supplemented by 15–20 minutes of daily home practice. For individuals primarily experiencing low mood or lack of motivation, prioritize aerobic exercise. This promotes natural mood elevation by releasing endorphins and regulating neuroendocrine function. 3 weekly sessions of moderate-intensity outdoor brisk walking or group aerobic classes to gain dual benefits from both exercise and social interaction.

For individuals with pronounced post-traumatic stress reactions or disease-related anxieties, mind-body practices serve as first-line interventions, especially when integrated with self-hypnosis or mindfulness-based cognitive therapy. Such approaches help reframe perceptions of illness and symptoms, alleviating psychological burdens.

Tiered Recommendations Based on Comorbid Conditions, for individuals with metabolic conditions such as cardiovascular disease or diabetes, aerobic exercise should be prioritized as it simultaneously improves metabolic markers and cardiovascular function. Start at low intensity under professional guidance, gradually progressing to moderate intensity while monitoring physical responses during exercise. For individuals experiencing joint pain, mobility limitations, or impaired balance, home-based mind-body exercises such as seated Tai Chi, chair yoga, or mindful breathing practices are recommended. These interventions offer high safety profiles and can alleviate pain-related distress by enhancing proprioceptive awareness and body consciousness. For individuals with multiple comorbidities and complex functional status, adopt a progressive intervention strategy characterized by low intensity, short duration, and high frequency. Begin with 2–3 sessions per week of 15-minute mind-body exercises, gradually increasing duration and frequency. Introduce low-intensity aerobic exercise as tolerated.

Tiered Recommendations Based on Treatment Phase, for individuals undergoing adjuvant therapies, interventions centered on mind-body practices are recommended due to their controllable intensity and adaptability. Mindful breathing, guided imagery, and gentle stretching exercises help alleviate treatment-related side effects and improve sleep quality. For patients who have completed primary treatment and entered the recovery phase, aerobic exercise and resistance training can be gradually introduced to promote overall functional recovery. A combined model of mind-body exercises and aerobic exercise is recommended, such as two yoga sessions per week combined with two brisk walking sessions.

Regardless of the intervention strategy chosen, the following key points should be observed in clinical practice: First, intervention intensity should follow the principle of low-to-high, step-by-step progression, particularly for elderly patients with long-term physical inactivity. Second, group intervention formats provide valuable social support, enhancing compliance and long-term adherence. Third, digital intervention platforms offer viable alternatives for mobility-impaired individuals and should be considered in clinical practice. Finally, regularly assessing symptom changes and intervention tolerance to promptly adjust protocols is key to achieving personalized, precision rehabilitation.
